# Changes in Electric Properties of Human Breast Cancer Cells

**DOI:** 10.1007/s00232-012-9516-5

**Published:** 2012-11-08

**Authors:** Izabela Dobrzyńska, Elżbieta Skrzydlewska, Zbigniew A. Figaszewski

**Affiliations:** 1 Institute of Chemistry, University in Białystok, Al. Piłsudskiego 11/4, 15-443 Białystok, Poland; 2 Department of Analytical Chemistry, Medical University of Białystok, Mickiewicza 2, 15-230 Białystok, Poland; 3 Laboratory of Electrochemical Power Sources, Faculty of Chemistry, University of Warsaw, Pasteur St. 1, 02-093 Warsaw, Poland

**Keywords:** Surface charge density, Breast cancer cell (MDA-MB-231, MCF-7), Fibroblast, Lipid peroxidation

## Abstract

Studies of the electrical surface properties of biological cells have provided fundamental knowledge about the cell surface. The change in biological functions of cells may affect the surface properties and can be detected by electrokinetic measurements. The surface density of fibroblasts and breast cancer cells (MDA-MB-231 and MCF-7) as a function of pH was measured by electrophoresis. The interaction between solution ions and the breast cancer cell or fibroblast surface was described by a four-component equilibrium model. The agreement between the experimental and theoretical charge variation curves of the breast cancer cells and fibroblasts was good at pH 2.5–9. The extent of fibroblast and breast cancer cell lipid peroxidation was estimated by HPLC measurement of the malondialdehyde level. The acid (*C*_TA_) and basic (*C*_TB_) functional group concentrations and the average association constant with hydroxyl (*K*_BOH_) ions values of the breast cancer cell membranes were higher than in normal cells, while the average association constant with hydrogen (*K*_AH_) value was smaller. The level of lipid peroxidation products was higher in breast cancer cells than in normal cells.

## Introduction

The electric charge of cell membranes of mammals is negative at physiological pH (Benga and Holmes 1984). Conservation of membrane structure and of its proper surface charge is crucial in processes in which the membrane is involved. Among them the most important are transport of metabolism substrates and products by ion pumps, membrane carriers and channels as well as information transmission (Nałęcz 1989).

Any perturbation in the action of the cell is manifested by variations in the action of the cell membrane, i.e., in its electric layer. An essential property of the electric double layer is its electric charge, which can be altered by various xenobiotics or by metabolic transformations. For this reason, studies of electric charge can furnish much information on the equilibrium existing within the membrane and between the membrane and its environment, both in physiological and in nonphysiological conditions (Szachowicz-Petelska et al. 2012; Gennis 1989; Dołowy 1984). Cell membrane charge increases during tumorigenesis and decreases during necrosis (Dołowy 1984). Determining the electric charge of the membrane as a function of environmental pH, acid (*C*_TA_) and basic (*C*_TB_) functional group concentrations and their average association constants with hydrogen (*K*_AH_) or hydroxyl (*K*_BOH_) ions allows monitoring of changes caused by cancer transformation (Dobrzyńska et al. 2006).

It is well known that malignant neoplastic cells are different in their surface properties from their normal counterparts. Unusual cell-to-cell interactions in malignant cells are the most important behavior for distinguishing them from their normal counterparts and for determining the prognosis of patients suffering from cancer.

This work continues the study of electrical property changes of cell membranes under the influence of cancer transformation (Dobrzyńska et al. 2005; Szachowicz-Petelska et al. 2010). We examined changes in the electric charge of cell membranes caused by cancer transformation in an in vitro study.

Cancerous cell lines are an excellent way for examining the mechanisms of carcinogenesis, and research using vitro cultures is bringing significant benefit. Therefore, the purpose of this work was to determine the electrical properties of the membrane and the level of lipid peroxidation products of human breast cancer cells (MDA-MB-231 and MCF-7) and normal cells (fibroblasts). In our opinion, the quantitative description of cell membrane surface properties can help in interpreting and understanding the processes that take place on biological membrane surfaces during cancer transformation.

## Theory

The model, which has been presented in full detail in a previous study (Dobrzyńska et al. 2006), assumes that the dependence of the surface charge density of the cell membrane on the pH of an electrolyte solution can be described with the help of four equilibria. There are two equilibria of negative groups, with sodium and hydrogen ions, and two equilibria of the positive groups, with hydroxide and chloride ions. H^+^, OH^−^, Na^+^ and Cl^−^ ions are adsorbed at the cell membrane (fibroblasts, MDA-MB-231, MCF-7), and the adsorption equilibria (Eqs. 1–4) can by presented in the following forms:

1 $$ A^{ - } + {\text{H}}^{ + } \Leftrightarrow A{\text{H}} $$

2 $$ A^{ - } + {\text{Na}}^{ + } \Leftrightarrow A{\text{Na}} $$

3 $$ B^{ + } + {\text{OH}}^{ - } \Leftrightarrow B{\text{OH}} $$

4 $$ B^{ + } + {\text{Cl}}^{ - } \Leftrightarrow B{\text{Cl}} $$

The association constants of the H^+^, Na^+^, OH^−^ and Cl^−^ ions with functional groups are expressed by the following equations:

5 $$ K_{\text{AH}} = \frac{{a_{\text{AH}} }}{{a_{{{\text{A}}^{ - } }} \cdot a_{{{\text{H}}^{ + } }} }} $$

6 $$ K_{\text{ANa}} = \frac{{a_{\text{ANa}} }}{{a_{{{\text{A}}^{ - } }} \cdot a_{{{\text{Na}}^{ + } }} }} $$

7 $$ K_{BOH} = \frac{{a_{\text{BOH}} }}{{a_{{{\text{B}}^{ + } }} \cdot a_{{{\text{OH}}^{ - } }} }} $$

8 $$ K_{\text{BCl}} = \frac{{a_{\text{BCl}} }}{{a_{{{\text{B}}^{ + } }} \cdot a_{{{\text{Cl}}^{ - } }} }} $$

where *K*_AH_, *K*_ANa_, *K*_BOH_ and *K*_BCl_ are association constants; $a_{{{\text{A}}^{ - } }}$, $a_{\text{AH}}$, $a_{\text{ANa}}$, $a_{{{\text{B}}^{ + } }}$, $a_{\text{BOH}}$ and $a_{\text{BCl}}$ are surface concentrations of the corresponding groups on the membrane surface; and $a_{{{\text{H}}^{ + } }}$, $a_{{{\text{Na}}^{ + } }}$, $a_{{{\text{OH}}^{ - } }}$ and $a_{{{\text{Cl}}^{ - } }}$ are the corresponding concentrations in solution.

Surface charge density (*δ*) is expressed as follows:

9 $$ \delta = (a_{{{\text{B}}^{ + } }} - a_{{{\text{A}}^{ - } }} ) \cdot F $$

where $F = 96,487$ $\left[ {\frac{C}{\text{mol}}} \right]$ is the Faraday constant.

Functional group concentration balances are expressed as follows:

10 $$ C_{\text{TA}} = a_{{{\text{A}}^{ - } }} + a_{\text{AH}} + a_{\text{ANa}} $$

11 $$ C_{\text{TB}} = a_{{{\text{B}}^{ + } }} + a_{\text{BOH}} + a_{\text{BCl}} $$

where $C_{\text{TA}}$ is the total surface concentration of acidic groups and $C_{\text{TB}}$is the total surface concentration of basic groups.

Elimination of $a_{{{\text{A}}^{ - } }}$, $a_{\text{AH}}$, $a_{{{\text{B}}^{ + } }}$ and $a_{\text{BOH}}$ values from the above equation yields the following formula:

12 $$ \frac{\delta }{F} = \frac{{C_{\text{TB}} \cdot a_{{{\text{H}}^{ + } }} }}{{a_{{{\text{H}}^{ + } }} (1 + K_{\text{BCl}} \cdot a_{{{\text{Cl}}^{ - } }} ) + K_{\text{BOH}} \cdot K_{\text{w}} }} - \frac{{C_{\text{TA}} }}{{K_{\text{AH}} \cdot a_{{{\text{H}}^{ + } }} + K_{\text{ANa}} \cdot a_{{{\text{Na}}^{ + } }} + 1}}, $$

It is difficult to solve Eq. 12 and determine the *K*_AH_, *K*_ANa_, *K*_BOH_ and *K*_BCl_ constants. In cases of high or low hydrogen ion concentrations, Eq. 12 can be simplified to a linear equation. In the range of high H^+^ concentrations, the numerator of each term in Eq. 12 can be divided by the denominator, leaving two initial terms only. These operations yield the linear equation in the $a_{{{\text{H}}^{ + } }}$and $\frac{\delta }{F}a_{{{\text{H}}^{ + } }}$ coordinate system:

13 $$ \frac{\delta }{F}a_{{{\text{H}}^{ + } }} = \frac{{C_{\text{TB}} }}{{1 + K_{\text{BCl}} \cdot a_{{{\text{Cl}}^{ - } }} }} \cdot a_{{{\text{H}}^{ + } }} - \left( {\frac{{K_{\text{BOH}} \cdot K_{\text{w}} \cdot C_{\text{TB}} }}{{(1 + K_{\text{BCl}} \cdot a_{{{\text{Cl}}^{ - } }} )^{2} }} + \frac{{C{}_{\text{TA}}}}{{K_{\text{AH}} }}} \right) $$

In graphical representation, the slope and the intercept can be easily extracted. At low H^+^ ion concentration Eq. 12 simplifies to

$$ \frac{\delta }{F} = \frac{{C_{TB} \cdot a_{{H^{ + } }} }}{{K_{BOH} \cdot K_{w} + a_{{H^{ + } }} (1 + K_{BCl} \cdot a_{{Cl^{ - } }} )}} - \frac{{C_{TA} }}{{K_{ANa} \cdot a_{{Na^{ + } }} + 1 + K_{AH} \cdot a_{{H^{ + } }} }} $$

The numerator of each term should be divided by the denominator, leaving two initial terms only. These operations yield a linear equation in the $a_{{{\text{H}}^{ + } }}^{ - 1}$ and $\frac{\delta }{F}a_{{{\text{H}}^{ + } }}^{ - 1}$ coordinate system:

14 $$ \frac{\delta }{F}a_{{{\text{H}}^{ + } }}^{\_1} = \frac{{ - C_{\text{TA}} \cdot a_{{{\text{H}}^{ + } }}^{ - 1} }}{{1 + K_{\text{ANa}} \cdot a_{{{\text{Na}}^{ + } }} }} + \left( {\frac{{C_{\text{TB}} }}{{K_{\text{BOH}} \cdot K_{\text{w}} }} + \frac{{K_{\text{AH}} \cdot C{}_{\text{TA}}}}{{(1 + K_{\text{ANa}} \cdot a_{{{\text{Na}}^{ + } }} )^{2} }}} \right) $$

In graphical representation, the slope and the intercept can be easily extracted.

The coefficients estimated from linear regression can be used to determine $C_{\text{TA}}$, $C_{\text{TB}}$, *K*_AH_ and *K*_BOH_. The points included in the regression must be carefully selected, in both high and low pH ranges. Defining the value of these parameters permits the calculation of the theoretical cell membrane surface charge from Eq. 12 for comparison to experimental data.

## Materials and Methods

### Cell Culture

Normal human skin fibroblasts, CRL-1474, and two types of breast cancer cells, MCF-7 and MDA-MB-231 (derived by the American Type Culture Collection, Manassas, VA), were maintained in DMEM containing 10 % fetal bovine serum, 50 U/ml penicillin and 50 μg/ml streptomycin in a humidified atmosphere of 5 % CO_2_ at 37 °C. Suspension of cells (1 × 10^6^ cells/ml) in 6 ml of culture medium were incubated with or without the test compounds in cell culture plates.

### Lipid Peroxidation

The extent of lipid peroxidation in cells was assayed as malondialdehyde (MDA) levels. MDA level was estimated as a condensation product reaction of thiobarbituric acid (TBA) with MDA that was separated by HPLC. We added 0.75 ml phosphate acid solution (0.44 M) and 0.25 ml freshly prepared TBA solution (42 mM) to 0.5 ml diluted lysate cells, and the mixture was incubated for 60 min at 100 °C. After cooling down, 0.5 ml of the above mixture was neutralized with a 0.5-ml 1 M methanol–1 M NaOH mixture (45.5:4.5, v:v). After centrifugation, 30 µl of solution was injected in the chromatographic column (RP18). Separation was carried out with isocratic elution (40 % methanol and 60 % phosphate buffer, pH 7.0). Detection with a spectrofluorimetric detector (*λ*_excitation_ = 532 nm, *λ*_emission_ = 553 nm) was done. The concentration of MDA was expressed in nanomoles TBA-rs per milliliter cell lysate.

### Electrochemical Method

In order to determine the surface charge density of cell membranes, cells were suspended in 0.015 M NaCl and put into the measuring vessel; then, electrophoretic mobility was measured using the Zetasizer Nano ZS apparatus (Malvern Instruments, Malvern, UK). Measurements were carried out as a function of pH.

Surface charge density was determined using the equation *σ* = *ηu*/*d*, where *u* is electrophoretic mobility, *η* is viscosity of solution and *d* is diffuse layer thickness (Krysiński and Tien 1986).

The diffuse layer thickness was determined from the formula (Barrow 1996) $d = \sqrt {\frac{{\varepsilon \varepsilon_{0} RT}}{{2F^{2} I}}}$, where *R* is the gas constant, *T* is the temperature, *F* is the Faraday number, *I* is the ionic strength of 0.9 % NaCl and *ε*_0_ is the permeability electric medium.

### Statistical Analysis

The data obtained in this study are expressed as mean ± SD. The data were analyzed using standard statistical analyses, one-way ANOVA with Scheffe’s *F* test for multiple comparisons to determine the significance between different groups. *p* < 0.05 was considered significant.

## Results and Discussion

Normal cells possess the ability to communicate information inside themselves and between other cells. The coordination of information by the cells of the body is involved in the regulation and integration of cellular functions and cell growth. When cancer arises, cancer cells are no longer regulated by the normal control mechanisms. Cancerous cells also possess other features that are different from normal proliferating cells. Normal cells are well organized in their growth, form strong contacts with their neighbors and stop growing when they repair the area of injury due to contact inhibition with other cells. Cancer cells are more easily detached and do not exhibit contact inhibition of their growth. Cancer cells become independent of normal tissue signaling and growth-control mechanisms. In a sense cancer cells become desynchronized from the rest of the body.

**Fig. 1:**
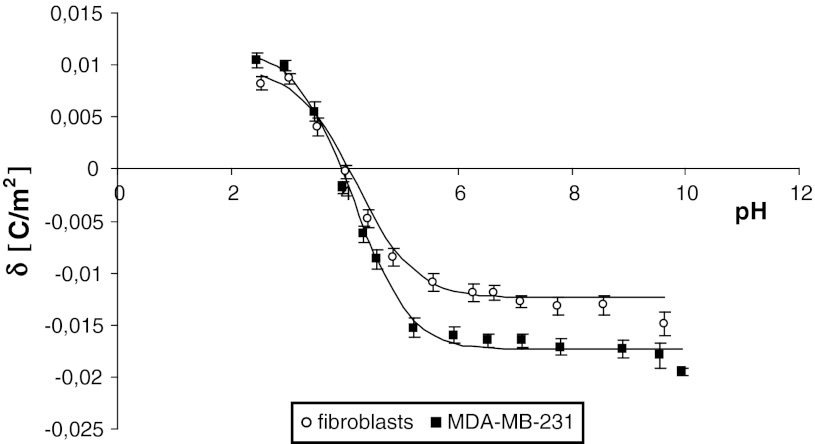
Dependence of surface charge density of fibroblasts and breast cancer cell (MDA-MB-231) membranes on pH (experimental values are marked by *points* and theoretical ones by *line*)

**Fig. 2:**
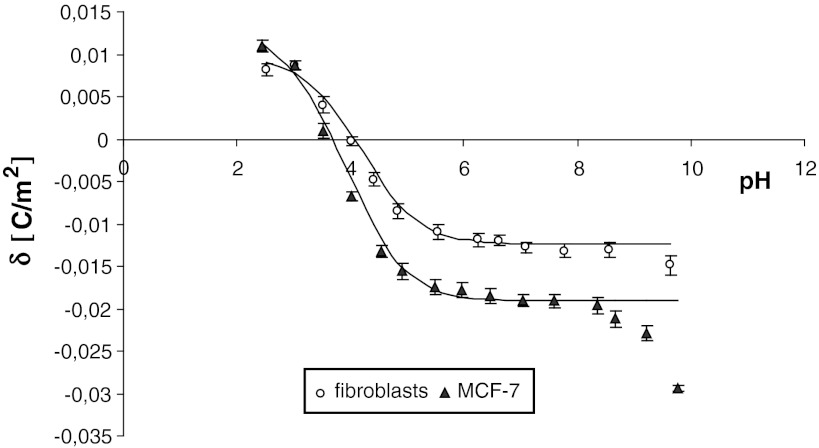
Dependence of surface charge density of fibroblasts and breast cancer cell (MCF-7) membranes on pH (experimental values are marked by *points* and theoretical ones by *line*)

The surface charge density (experimental and theoretical) of the cell membrane as a function of pH are presented in Figs. 1 and 2. The former are indicated by points, and the latter are indicated by curves. It can be seen in the figures that the surface charge density dependence of MDA-MB cells, MCF-7 cells and fibroblasts on pH is similarly shaped for all studied cells. There is an increase in positive surface charge density of the cells at low pH values until a plateau is reached. At high pH values, the negative charge of the cells also increases, reaching a plateau.

The negative charge at high pH values as well as the positive charge at low pH values of human breast cancer cells are higher than those in fibroblasts, with a shift of isoelectric point of the membrane to low pH values (Figs. 1, 2).

Mathematical calculations based on the four equilibria model (presented above, theory), describing adsorption of electrolyte ions on a cell membrane surface, enabled quantitative evaluation of the membrane characterizing parameters. The total concentrations of functional acidic (*C*_TA_) and basic (*C*_TB_) groups on breast cancer cell as well as fibroblast surfaces and their average association constants with hydrogen (*K*_AH_) and hydroxyl (*K*_BOH_) ions were calculated based on Eqs. 13 and 14. The *C*_TA_, *C*_TB_, *K*_AH_ and *K*_BOH_ constants resulting from determination were substituted into Eq. 12, yielding the theoretical curve. Typical experimental points and the theoretical curve are presented in Figs. 1 and 2. It can be observed that the theoretical and experimental surface charge density values agree. The *C*_TA_, *C*_TB_ and *K*_BOH_ values of the cell membranes modified by cancer transformation were higher than in normal cells, while the *K*_AH_ value was smaller (Table 1).

**Table 1:** C_TA_, C_TB_, *K*_AH_ and *K*_BOH_ of fibroblasts and breast cancer cells (MDA-MB-231, MCF-7) membranes

Groups	Parameters			
	*C*_TA_ (10^−7^ mol/m^2^)	*C*_TB_ (10^−7^ mol/m^2^)	*K*_AH_ (m^3^/mol)	*K*_BOH_ (10^7^ m^3^/mol)
Fibroblasts	1.23 ± 0.08	0.96 ± 0.06	32.80 ± 0.90	1.68 ± 0.08
MCF-7	1.73 ± 0.10*	1.16 ± 0.09*	26.81 ± 1.10*	2.92 ± 0.12*
MDA-MB-231	1.90 ± 0.12*	1.31 ± 0.10*	18.8 ± 1.08*	4.97 ± 0.18*

* *p* < 0.05, compared with fibroblasts

It has been demonstrated that cancer transformation causes modifications in the lipid bilayer membrane (Punnonen et al. 1989; Szachowicz-Petelska et al. 2012). An increase in the content of phospholipids is observed in human breast cancer cells (Sakai et al. 1992; Punnonen et al. 1989; Podo et al. 2007). Increased phospholipid content may result from enhanced cell membrane synthesis related to accelerated neoplasm cell replication (Ruiz-Cabello and Cohen 1992). Increased amount of phospholipids results in a higher amount of functional groups: amino, carboxy and phosphate groups. In acid medium (low pH) the charge of phospholipids is mainly due to amino groups, whereas in basic medium (high pH) it is due to carboxy and phosphate groups. Increased amount of phospholipids can increase the surface density of negatively charged groups of breast cancer cell membranes at high pH values and that of positively charged ones at low pH.

Hypoxia/reoxygenation and acidity induced exposure of anionic phospholipids, most likely phosphatidylserine and phosphatidylethanolamine (Zhao et al. 1998; Ran et al. 2002). According to previous studies both hypoxia and acidity can exist in a tumor. In particular, hypoxia represents an important cellular stressor that can trigger a survival program by which cells attempt to adapt to a new environment. Typically, these adaptations will largely affect cell metabolism and/or stimulation of oxygen delivery (Bos et al. 2004).

Cell membrane charge is also affected by sialic acid present in glycolipids and glycoproteins. It has been supposed that sialic acid also influences the surface concentration of acid and basic groups as well as the association constants of positive and negative groups during cancer transformation. Increased sialic acid content in glycolipids and glycoproteins has been confirmed by literature data (Narayanan 1994; Jakielaszek et al. 1986). Increased sialic acid content can provoke increased surface concentration of acid groups.

Table 2 shows changes in the level of final lipid peroxidation product MDA measured by HPLC as a TBA derivative. The level of MDA was significantly higher in breast cancer cells (MDA-MB-231, MCF-7) than in normal cells (Table 2).

**Table 2:** Levels of final lipid peroxidation product malondialdehyde (MDA, nmol/mg) in fibroblasts and breast cancer cells (MDA-MB-231, MCF-7)

Fibroblasts	MDA-MB	MCF-7
1.00 ± 0.05	1.39 ± 0.04*	10.57 ± 0.42*

* *p* < 0.05 compared with fibroblasts

Membrane lipids and proteins can also be modified by reactive oxygen species (ROS) appearing as the result of cancer transformation (Gago-Dominguez et al. 2007; Skrzydlewska et al. 2005). ROS provoke the so-called lipid peroxidation. As a consequence, phosphatidylserine molecules, physiologically present on the inner side of the membrane, become exposed on the outer side (Tyurina et al. 2000). Exposing the phosphatidylserine molecule to the outer side of the membrane makes an additional negatively charged group appear there. It provokes an increase in acid group surface concentration, as confirmed in this work (Table 1). The presence of additional functional groups of phosphatidylserine can result in a decreased association constant of negatively charged groups of the membrane and an increased association constant of positively charged ones because the *K*_AH_ value is lower and the *K*_BOH_ value is higher than those of the original cell membrane groups (Dobrzyńska et al. 2007).

The reaction of aldehydes produced during lipid peroxidation with amino acid residues of proteins might lead to their oxidative modification. In this process, the final products of lipid peroxidation, such as MDA as well as other products resulting from polyunsaturated fatty acid damage, could cause protein breakdown (Esterbauer et al. 1991).

The ultimate consequence of oxidative modification of proteins can be their aggregation or fragmentation (Davies et al. 1987; Du and Gebicki 2002). Protein fragmentation creates increasing functional groups, both acidic and basic. The process can also expose membrane phospholipid functional groups hitherto screened by proteins. The changes can yield higher *C*_TA_ values and lower *C*_TB_ values, as confirmed by this work.

The data suggest that the electrical properties of breast cancer cells are different from those of normal cells. The constants *C*_TA_, *C*_TB_, *K*_AH_ and *K*_BOH_ are suited for monitoring the changes caused by cancer transformation. Therefore, an evaluation of the parameters characterizing cancer cell membranes may be an important consideration in future studies of cancer biology.
